# Prevalence and factors associated with overweight and central obesity among adults in the Eastern Sudan

**DOI:** 10.1371/journal.pone.0232624

**Published:** 2020-04-30

**Authors:** Saeed M. Omar, Zainab Taha, Ahmed Ali Hassan, Osama Al-Wutayd, Ishag Adam

**Affiliations:** 1 Faculty of Medicine, Gadarif University, Gadarif, Sudan; 2 College of Natural and Health Sciences, Zayed University, Dubai, United Arab Emirates; 3 Faculty of Medicine, University of Khartoum, Khartoum, Sudan; 4 Department of Family and Community Medicine, Unaizah College of Medicine and Medical Sciences, Qassim University, Unaizah, Saudi Arabia; 5 Department of Obstetrics and Gynecology, Unaizah College of Medicine and Medical Sciences, Qassim University, Unaizah, Saudi Arabia; Makerere University School of Public Health, UGANDA

## Abstract

**Background:**

A global epidemic of obesity has been documented, particularly among African countries. While central obesity and overweight have been reported for many countries, very limited information exists about the prevalence of these health problems in Sudan, and these data are nonexistent for Eastern Sudan. The present study aimed to determine the prevalence of obesity and central obesity, as well as the factors associated with both, among adults in Gadarif, Eastern Sudan.

**Methods:**

A cross-sectional study was conducted in Gadarif, Eastern Sudan, during the period of January through May 2018. Sociodemographic and health characteristics data were collected through a questionnaire. Body mass index (BMI) and waist circumference (WC) were measured using the standard methods. Both descriptive and inferential statics were applied to analyze the data.

**Results:**

A total of 594 adults participated in the study; 70.4% of them were female. The mean (standard deviation) age was 44.98 (16.64) years. Of the 594 enrolled participants, 33.7%, 7.4%, 26.8%, and 32.2% were normal weight, underweight, overweight, and obese, respectively. The prevalence of central obesity was (67.8%). Approximately, one-third of the participants (29.29%) were obese and had central obesity. In the multinomial regression, being married was the main risk factor associated with overweight, and older age, female sex, being married and hypertension were significantly associated with obesity. In the binary regression, the main risk factors associated with central obesity were female sex and being married.

**Conclusion:**

The prevalence rates of both obesity and central obesity among the study participants were high. Older age and hypertension were only associated with obesity. Obesity and central obesity were significantly associated with female sex and being married. This study provided valuable baseline information to develop appropriate strategies for the prevention and control of obesity in Eastern Sudan.

## Introduction

According to the World Health Organization (WHO), obesity is considered a major health problem in both developed and developing countries [1]. Excess weight is one of the leading causes of morbidity and mortality, and it is increasing exponentially worldwide [2,3]. Currently, over half a billion adults are considered obese [4]. The documented global epidemic of obesity has been increasing at an alarming rate, particularly among African countries [5]. The prevalence of overweight and obesity among adults in the Eastern Mediterranean Region ranges from 25% to 81.9% [6]. The effect of obesity on health has been well documented in the literature; it is a disease in its own right as well as a risk factor for several metabolic disorders including diabetes, hypertension, dyslipidemia, cardiovascular disease and even some cancers [7]. Both overweight and obesity were associated with a significantly increased risk of coronary heart disease and stroke [8], diabetes mellitus and hypertension [9]. While overall obesity is well documented to be a significant problem with major health risks, the distribution of body fat is of great importance in determining this risk [10,11]. Therefore, abdominal fat deposition measured by waist circumference (WC) has been suggested as a better indicator of obesity in relation to metabolic syndrome, type 2 diabetes, and cardiovascular diseases than body mass index (BMI) [12]. Various risk factors, such as female sex, have been reported to be associated with both obesity and central obesity [13]. In low-income countries, obesity mostly affects middle-aged adults (especially women) from wealthy, urban environments, whereas in high-income countries, it affects both sexes and all ages, but it is disproportionately more common in disadvantaged groups [5]. Central obesity has been associated with an increase in age and female sex [13]. In the WHO regions for Africa, Eastern Mediterranean and South East Asia, women had roughly double the obesity prevalence of men [8]. Previous studies have found that overweight/obesity and central obesity were important risk factors for hypertension and diabetes [14]. Importantly, it is predicated that obesity, a medical condition characterized by an abnormal fat accumulation that is detrimental to health, will put tremendous pressure on the health systems of many Sub-Sahara African countries [8]. Although the nationwide data on the prevalence of overweight are available for some developing countries [2,14–17], very limited studies have been reported for Sudan [9]. Accurate information regarding the prevalence of overweight and obesity is important for appropriate public health responses. Therefore, the current study aimed to estimate the prevalence of obesity and central obesity among adults, as well as the associated factors, in Gadarif, Eastern Sudan.

## Materials and methods

A multistage sampling study was conducted in Gadarif, Eastern Sudan. Initially, four Mahlyiat (lowest administrative unit in Sudan) were selected from the eleven Mahlyiat of the Gadarif by simple random sampling. Then, the total sample size of 590 subjects was distributed among the four Mahlyiat according to size allocation of each Mahlyiat. Finally, the selected subjects were then chosen with a lottery method among the households. Trained medical officers interviewed the participants during the period of January through May 2018. All eligible participants were invited to participate in the study. Information about the study was given to the participants by the medical officers. Five hundred and ninety-four adults who met the inclusion criteria of age ≥20 years and not pregnant (female participant) were interviewed by medical officers using a structured questionnaire. A pilot study was conducted and face validity was determined by the principal investigator to validate the questionnaire prior to distributing it. The collected data included sociodemographic variables (e.g., age, sex, education, occupation), behavioral characteristics (e.g., smoking, alcohol drinking), and anthropometric measurements (weight, height, and WC). Blood pressure and blood sugar levels were measured. Weight and height were measured to the nearest 0.1 kg and 0.1 cm, respectively, with the participants in lightweight clothing and without shoes. BMI was computed by the formula: weight in kilograms divided by height in meters squared. Furthermore, the BMI was categorized according to the WHO classification as underweight (<18.5 kg/m^2^), normal weight (18.5–24.9 kg/m^2^), overweight (25.0–29.9 (kg/m^2^) and obese (≥30.0 kg/m^2^) [1].

WC was measured at the midpoint between the lower margin of the least palpable rib and the top of the iliac crest using a stretch-resistant tape that provided a constant 100 g of tension. As there are no specific guidelines for waist circumference for Sub-Saharan Africans, the European cut off to interpret the WC measurements was used as per the WHO and International Diabetes Federation recommendations: ≥94 cm and ≥80 cm for males and females, respectively [18]. A sample size of 594 participants was calculated based on the previously reported incidence of both overweight and obesity (56.1%) among the Sudanese population [9] and a design effect of 1.5. This sample size had 80% power with a precision of 5% and an assumption that 10% of the participants would either not respond or have incomplete data. The study received ethical approval from the Research Board at the Faculty of Medicine, University of Gadarif, Sudan; the reference number is 2017/13. Written informed consent was obtained from all the enrolled participants after explaining to them the purpose of the study, the voluntary nature of their participation, their freedom to withdraw from the study at any time, and the confidentiality of the collected data. Data were entered and analyzed using the Statistical Package for Social Sciences (SPSS) version 20.0 for Windows (IBM Corp, New York, United States). The results were illustrated in tables and text by calculating the mean (M) and standard deviation (SD) for continuous variables and frequencies and proportions for categorical variables to describe the participants’ responses. T-tests and analysis of variance (ANOVA) were used to compare the continuous variables between participants with central obesity (yes, no) and among the four subgroups of the BMI (underweight, normal, overweight and obese), respectively. Proportions were compared by chi-square test for both the BMI subgroups and central obesity. Variables with a P-value of < 0.20 on the chi-square test were further analyzed by logistic regressions (multinomial and binary for BMI groups and central obesity, respectively). The underweight, smoking and alcohol usage categories were found to have small numbers and were removed from the model. Binary logistic regression (backward LR) was performed for the central obesity outcome. Odds ratios [ORs], adjusted odds ratios [AORs], and 95% confidence intervals [CIs] were calculated and variables with a P-value < 0.05 were considered statistically significant.

## Results

A total of 594 adults participated in the study. The mean (SD) age of the participants was 44.98 (16.64) years. The sociodemographic characteristics are described in (Table 1). Out of the 594 participants, 44 (7.4%), 200 (33.7%), 159 (26.8%), and 191 (32.2%) were underweight, normal weight, overweight and obese, respectively. Four hundred–three (67.8%) participants had central obesity. Out of the total sample of 594 participants, 174 participants (29.29%) were obese in addition to having central obesity.

**Table 1:** Sociodemographic characteristics of the studied participants in Gadarif, Eastern Sudan (N = 594).

Characteristics		Number	Proportion
**Age group (years)**	<40	255	42.9
	≥40	339	57.1
**Sex**	Male	176	29.6
	Female	418	70.4
**Body mass index**	Underweight	44	7.4
	Normal	200	33.7
	Overweight	159	26.8
	Obese	191	32.2
**Central obesity**	Yes	403	67.8
	No	191	32.2
**Marital status**	Married	421	70.9
	Unmarried	173	29.1
**Education status**	< secondary level	259	43.6
	≥Secondary	335	56.4
**Occupation status**	Unemployed	397	66.8
	Employed	197	33.2
**Smokers**	Yes	15	2.5
	No	579	97.5
**Alcohol consumers**	Yes	2	0.3
	No	592	99.7
**Hypertension**	Yes	174	29.3
	No	420	70.7
**Diabetes mellitus**	Yes	118	19.9
	No	476	80.1

The sociodemographic characteristics among the different BMI subgroups are described in (Table 2).

**Table 2:** Sociodemographic characteristics among the different body mass index subgroups (N = 594) in Eastern Sudan.

Characteristics		Normal	Underweight	Overweight	Obese	P-value
		weight	(N = 44)	(N = 159)	(N = 191)	
		(N = 200)				
*Mean (SD) were compared using ANOVA*						
Age (years)		43.6 (18.3)	39.4 (16.8)	46.4 (16.53)	46.3 (14.4)	0.034
WC (cm)		85.9 (14.2)	69.9 (16.9)	90.7 (17.99)	102.1 (21.1)	< 0.001
*Number(proportions) were compared using Chis square test*						
**Age, years**	<40	98 (38.4)	27 (10.6)	64 (25.1)	66 (25.9)	0.002
	≥40	102 (30.1)	17 (5.0)	95 (28.0)	125 (36.9)	
**Sex**	Male	76 (43.2)	13 (7.4)	49 (27.8)	38 (21.6)	0.001
	Female	124 (29.7)	31 (7.4)	110 (26.3)	153 (36.6)	
**Marital status**	Married	116 (27.6)	22 (5.2)	117 (27.8)	166 (39.4)	< 0.001
	Unmarried	84 (48.6)	22 (12.7)	42 (24.3)	25 (14.5)	
**Education status**	< Secondary level	85 (32.8)	10 (3.9)	79 (30.5)	85 (32.8)	0.016
	≥Secondary	115 (34.3)	34 (10.1)	80 (23.9)	106 (31.6)	
**Occupation status**	Unemployed	130 (32.7)	26 (6.5)	111 (28.0)	130 (32.7)	0.520
	Employed	70 (35.5)	18 (9.1)	48 (24.4)	61 (31.0)	
**Smokers**	Yes	10 (66.70	1 (6.7)	2 (13.3)	2 (13.3)	0.052
	No	190 (32.8)	43 (7.4)	157 (27.1)	189 (32.6)	
**Alcohol consumers**	Yes	1 (50.0)	1 (50.0)	0 (0.0)	0 (0.0)	0.100
	No	199 (33.6)	43 (7.3)	159 (26.9)	191 (32.3)	
**Hypertension**	Yes	53 (30.5)	15 (8.6)	40 (23.0)	66 (37.9)	0.164
	No	147 (35.0)	29 (6.9)	119 (28.3)	125 (29.8)	
**Diabetes mellitus**	Yes	40 (33.9)	9 (7.6)	29 (24.6)	40 (33.9)	0.937
	No	160 (33.6)	35 (7.4)	130 (27.3)	151 (31.7)	

There was no significant difference in occupation status, smoking status, alcohol consumption, or diabetes mellitus between the BMI groups. The results of the multinomial logistic regression revealed that the main factor significantly associated with overweight was being married (OR 1.92 (95% CI 1.21, 3.04)) and with obesity were age ≥40 years (OR 1.83 (95% CI 1.16, 2.90)), female sex (OR 2.43 (95% CI 1.49, 3.98)), being married (OR 4.37 (95% CI 2.60, 7.35)), and hypertension (OR 1.64(95% CI 1.02, 2.63)) (Table 3).

**Table 3:** Multinomial logistic regression analysis of factors associated with overweight and obesity*.

Characteristics		Overweight (n = 159)		Obese (n = 191)	
		OR 95% (CI)	P-value	OR 95% (CI)	P-value
**Age, years**	≥40	1.29 (0.82, 2.04)	0.271	1.83 (1.16, 2.90)	0.010
	<40	Reference			
**Sex**	Female	1.24 (0.78, 1.97)	0.363	2.43 (1.49,3.98)	< 0.001
	Male	Reference			
**Marital status**	Married	1.92 (1.21, 3.04)	0.005	4.37 (2.60,7.35)	< 0.001
	Unmarried	Reference			
**Education status**	< Secondary level	1.14 (0.72, 1.81)	0.574	0.82 (0.52,1.10)	0.392
	≥Secondary	Reference			
**Hypertension**	Yes	1.06 (0.64, 1.74)	0.821	1.64 (1.02,2.63)	0.042
	No	Reference			

The sociodemographic characteristics of the participants with and without central obesity are shown in Table 4. There was no significant difference in hypertension or diabetes mellitus between the participants with central obesity and the participants without central obesity.

**Table 4:** Sociodemographic characteristics associated with central obesity (N = 594).

Characteristics		Central obesity	No central	p value
		(N = 403)	obesity (N = 191)	
*Mean (SD) were compared using the t-test*				
**Age (years)**		45.83 (16.43)	43.18 (16.96)	0.069
**BMI (kg/m2)**		29.22 (6.35)	23.61 (6.09)	< 0.001
*Number (proportions) were compared using the chi-square test*				
**Age, years**	o	161 (63.1)	94 (36.9)	0.033
	≥40	242 (71.4)	97 (28.6)	
**Sex**	Male	70 (39.8)	106 (60.2)	< 0.001
	Female	333 (79.7)	85 (20.3)	
**Marital status**	Married	314 (74.6)	107 (25.4)	< 0.001
	Unmarried	89 (51.4)	84 (48.6)	
**Education status**	< Secondary level	187 (72.2)	72 (27.8)	0.046
	≥Secondary	216 (64.5)	119 (35.5)	
**Occupation status**	Unemployed	290 (73.0)	107 (27.0)	< 0.001
	Employed	113 (57.4)	84 (42.6)	
**Smokers**	Yes	6 (40.0)	9 (60.0)	0.019
	No	39 7(68.6)	182 (31.4)	
**Alcohol consumers**	Yes	0 (0.0)	2 (100.0)	0.040
	No	403 (68.1)	189 (31.9)	
**Hypertension**	Yes	119 (68.4)	55 (31.6)	0.855
	No	284 (67.6)	136 (32.4)	
**Diabetes mellitus**	Yes	76 (64.4)	42 (35.6)	0.372
	No	327 (68.7)	149 (31.3)	

The factors significantly associated with central obesity were female sex (AOR 6.13 (4.12, 9.13)) and being married (AOR 2.93(1.95, 4.39)) (Table 5).

**Table 5:** Multivariable analysis for sociodemographic characteristics associated with central obesity.

Characteristics		Crude Odds Ratio	P- value	Adjusted Odds Ratio	P- value
		(95% CI)		(95% CI)	
**Age, years**	≥40	1.44 (0.94, 2.19)	0.090	-	-
	<40	reference			
**Sex**	Female	5.96 (3.82, 9.30)	<0.001	6.13 (4.12, 9.13)	<0.001
	Male	reference			
**Marital status**	Married	2.75 (1.82, 4.16)	<0.001	2.93 (1.95, 4.39)	<0.001
	Unmarried	reference			
**Education status**	< Secondary level	0.95 (0.62, 1.47)	0.823	-	-
	≥Secondary	reference			
**Occupation status**	Unemployed	1.10 (0.70, 1.74)	0.678	-	-
	Employed	reference			

## Discussion

As one of the first studies in Eastern Sudan that has investigated overweight and obesity, several results of great importance were found. This study provided valuable baseline information about the prevalence of overweight and obesity and the factors associated with these conditions. The overall prevalence of both overweight and obesity was 350 (59.0%), (26.8% overweight vs. 32.2% obese). Our result of the prevalence of overweight (59.0%) is higher than the prevalence of overweight in Uganda (17.8%) [15]. The prevalence rates of obesity and central obesity in the current study were higher than those previously reported in Northern part of Sudan (obesity 32.2% vs. 21.2% and central obesity 67.8% vs. 54.3%) [9]. This discrepancy could be attributed to two factors. The first factor was that the prevalence of overweight was higher in the previous study (34.9% vs. 26.8%), whereas the rate of overall obesity was higher in the current study (59.0% vs. 56.1%). The second was that more females were included in the current study compared to the previous one (70.4% vs. 59.1%). The higher number of females included in the current study could be due to their availability in the sampled households during the survey. Perhaps the males were out their houses during the study data collection times. On the other hand, the prevalence of overweight is more likely to be higher among men than women in most Eastern Mediterranean Region Countries [6]. The reverse (lower prevalence of overweight among men than in women) has been reported in Uganda [15]. The results of several studies conducted in different countries, including Sudan, have shown higher rates of obesity and central obesity among females compared to males [9,16]. For example, a higher rate of obesity in females was reported in Northern Sudan (26.3% vs. 13.8%) [9], and a similar higher rate of central obesity in females was reported in Uganda (19.5% vs. 1.3%) [16]. Similar to the current study, the prevalence of central obesity in South Africa was reported to be 67.0% [17]. In contrast to the prevalence in the present study, low rates of overall prevalence of overweight/obesity (29%) and central obesity (21%) were reported in India [14]. The present study showed that obesity was associated with older age, female sex, being married, and hypertension while central obesity was associated with female sex and being married. The present results revealed that older aged participants had almost double the risk of being obese (1.83 (1.16, 2.90)). This finding is on par with previous studies conducted in the Balearic Islands and Sudan [19,20]. The current results revealed that females were at almost two and a half (2.43 (1.49, 3.98)) and six times (6.13 (4.12, 9.13)) greater risk of obesity, and central obesity, respectively. In line with the current results, females in Northern Sudan [9] and Uganda [15,16], were at higher risk of obesity and central obesity. Consistent with the current results, the risk factors associated with central obesity in South Africa were female sex (11.1 (7.6, 16.2)) and being married (1.9 (1.3, 2.9)). Thus, these factors are of paramount importance as predictors of central obesity among the study participants [17]. Various studies conducted in the Middle East and Africa, including Sudan, showed that the higher prevalence of obesity in females than males can be attributed to less physical activity, rapid urbanization, less employment and cultural reasons [2,3,21]. The current study revealed that participants who were married were more likely to be obese (4.37 (2.60, 7.35)) and have central obesity (2.93 (1.95, 4.39)). The literature on the association between marital status and central obesity is inconsistent. Some researchers have reported similar positive associations between being married and weight gain [19,22,23]. However, others have reported different patterns among married women [24]. The linkage between marriage, obesity and central obesity has been suggested to be related to altered lifestyle practices such as little concern regarding body image after marriage and child birth [22]. The present analysis also revealed a consistent association between high blood pressure and obesity (1.64 (1.02, 2.63)). Consistent with the current results, obesity was reported in many countries, including Northern Sudan, as one of the leading risk factors for hypertension [9,14,25]. Obese older adults are at higher risk of comorbidities such as hypertension [26]. Unlike the previous study in Ghana [27], the current study showed that participants with low education were less likely to be underweight (0.40 (0.18, 0.92)). While the WHO endorses the classification of overweight and obesity by BMI, WC is also of great importance in evaluation of associated disease risks [1]. As expected, in the current study, central obesity steadily increased as the BMI increases. The rates of central obesity among the underweight, normal, overweight, and obese participants were 7/44 (15.9%), 103/200 (51.5%), 119/159 (74.8) and 174/191(91.1%), respectively. Similar to the current study, a strong correlation between obesity and central obesity was observed in other studies [9,17]. However, in China, hypertension, diabetes and hyperlipidemia were significantly associated with central obesity, even among adults with normal BMI (55.6%) [28]. Therefore, it is recommended to consider both BMI and central obesity as two separate indices during clinical evaluation.

Although the study provided valuable information, it has some limitations that need to be taken into consideration. The study focused on one geographical area of Sudan (Gadarif, Eastern Sudan); therefore, the results of this study cannot be generalized to the rest of the country. Moreover, the study failed to assess other variables such as the intensity, duration, and frequency of physical activity as well as cultural variables. These variables were found to be of great importance in the interpretation of the results reported in previous studies [2,21]. This was a cross-sectional study in which causal effects could not be dissected. There was a small number of smokers and alcohol users, which could be just an underestimation of the true situation because participants disliked revealing their habits. Likewise, females were not asked about their menopause status.

## Conclusions

The current study showed high prevalence rates of both obesity and central obesity among adults in Gadarif, which puts many populations at risk of developing associated metabolic complications in Eastern Sudan. Obesity and central obesity were significantly associated with female sex and being married. Older age and hypertension were associated only with obesity. These findings provide valuable information for developing interventions and for the formulation of short-term and long-term policies and strategies for the control and prevention of central obesity in Eastern Sudan.

## Supporting information

S1 Data (XLSX)
